# Intracortical inhibition in the soleus muscle is reduced during the control of upright standing in both young and old adults

**DOI:** 10.1007/s00421-016-3354-6

**Published:** 2016-03-22

**Authors:** Selma Papegaaij, Stéphane Baudry, János Négyesi, Wolfgang Taube, Tibor Hortobágyi

**Affiliations:** Center for Human Movement Sciences, University of Groningen, University Medical Center Groningen, Antonius Deusinglaan 1, 9713 AV Groningen, The Netherlands; Laboratory of Applied Biology, Faculty for Motor Sciences, Université Libre de Bruxelles, CP 640, route de Lennik 808, 1070 Brussels, Belgium; Department of Biomechanics, Faculty of Physical Education and Sport Sciences, Semmelweis University, Alkotás utca 44, Budapest, 1123 Hungary; Department of Medicine, University of Fribourg, Ch. du Musée 8, 1700 Fribourg, Switzerland; Department of Sport, Exercise and Rehabilitation, Northumbria University, Newcastle-upon-Tyne, NE1 8ST UK

**Keywords:** Aging, Balance, Short-interval intracortical inhibition, Transcranial magnetic stimulation, Peripheral nerve stimulation

## Abstract

**Purpose:**

In a previous study, we reported that a short-interval intracortical inhibition (SICI) decreases in old but not in young adults when standing on foam vs. a rigid surface. Here, we examined if such an age by task difficulty interaction in motor cortical excitability also occurs in easier standing tasks.

**Methods:**

Fourteen young (23 ± 2.7 years) and fourteen old (65 ± 4.1 years) adults received transcranial magnetic brain stimulation and peripheral nerve stimulation, while they stood with or without support on a force platform.

**Results:**

In the soleus, we found that SICI was lower in unsupported (35 % inhibition) vs. supported (50 %) standing (*p* = 0.007) but similar in young vs. old adults (*p* = 0.591). In the tibialis anterior, SICI was similar between conditions (*p* = 0.597) but lower in old (52 %) vs. young (72 %) adults (*p* = 0.030). Age and standing with or without support did not affect the Hoffmann reflex in the soleus.

**Conclusions:**

The current data suggest that the motor cortex is involved in standing control, and that its role becomes more prominent with an increase in task difficulty.

## Introduction

An increasing body of the literature supports the notion that the primary motor cortex (M1) is involved in the control of upright standing. For example, M1 excitability in the tibialis anterior and soleus is higher during normal compared with supported standing (Tokuno et al. 2009). Moreover, the amount of short-interval intracortical inhibition (SICI) that likely reflects the excitability of GABAergic inhibitory intracortical circuits (Di Lazzaro et al. 2000) is similar during a voluntary contraction of the soleus muscle while sitting and a postural contraction while standing (Soto et al. 2006). The involvement of M1 in balance control in young adults is further supported by the increase in corticomotoneuronal excitability in the soleus muscle after backward surface translation (Taube et al. 2006).

Recent studies reported an age-related increase in corticospinal excitability in the soleus muscle during the control of upright standing (Baudry et al. 2014a, b). This increase is accompanied by a decreased efficacy of Ia afferents to discharge spinal motor neurons (Baudry and Duchateau 2012; Baudry et al. 2014b; Koceja and Mynark 2000). These results imply an age-related increase in cortical contribution to control leg muscles during standing. Indeed, aging seems to influence the modulation of intracortical pathways, as indicated by the decrease in SICI from standing on a rigid surface to an unstable surface (foam mat) in old but not in young adults (Papegaaij et al. 2014). This reduction in SICI between balance tasks was associated with an increased center of pressure (CoP) velocity, highlighting the importance of intracortical circuits in controlling standing. An age-related increase in cortical control of standing could reflect a functional compensation for structural degeneration in the peripheral and central nervous system, increasing the need for a more adaptable control system.

The age-related difference in SICI modulation may be due to different motor control strategies between young and old. However, it may also be due to the task, i.e., standing on unstable surface, being more difficult for old than for young adults (Papegaaij et al. 2014). Therefore, our goal was to determine if there is also an age-related difference in the modulation of intracortical and spinal circuits during relatively simple balance tasks. Subjects stood naturally upright (unsupported standing) or while lightly touching a board at chest level (supported standing) to remove the need for the nervous system to control body sway. To examine SICI and intracortical facilitation (ICF), paired-pulse transcranial magnetic stimulation (TMS) was applied during the two conditions. To examine the efficacy of Ia afferents to activate spinal motor neurons, peripheral nerve stimulation was used.

We hypothesized an age-related decrease in SICI and a down modulation in SICI when standing unsupported vs. supported in old but not in young adults (Papegaaij et al. 2014). In contrast, we expected no age- or condition-related changes in ICF (Papegaaij et al. 2014). Based on previous data, we hypothesized greater down modulation of the Hoffmann reflex (H-reflex) from supported to unsupported standing in old compared with young adults (Baudry et al. 2014b; Tokuno et al. 2009).

## Materials and methods

### Participants

Twenty young adults and nineteen old adults volunteered for the study. In six young and five old adults, we stopped TMS data collection, because the stimulation intensity was above comfort threshold (>65 % maximum stimulator output). For the peripheral nerve stimulation (PNS), there were two young adults and four old adults in whom we could not evoke an H-reflex in the soleus. Therefore, TMS data from 14 young (age 23 ± 2.7 years, range 18–29, nine men) and 14 old (age 65 ± 4.1 years, range 60–76, eight men) adults, and PNS data from 18 young (age 23 ± 2.8 years, range 18–29, nine men) and 16 old (age 65 ± 4.1 years, range 60–76, eight men) adults were used in the statistical analyses. Subject characteristics were similar between those who finished and did not finish the experiments. Four young and one old subject were left-footed. None of the subjects had a history of or presented with neurological disorders, severe orthopedic disorders, suspicion of pregnancy, non-dental associated metal within the cranium, or took neuroactive drugs or drugs known to affect balance. To determine general cognitive function, physical activity in daily life, and lower extremity function, each subject completed the mini mental state examination (MMSE), the short questionnaire to assess health-enhancing physical activity (SQUASH), and the short physical performance battery (SPPB) (Table 1). Prior to their participation, subjects signed an informed consent document. The study was approved by the Medical Ethics Committee of the University Medical Center Groningen.

**Table 1:** Subject characteristics values are mean ± SD, unless denoted differently

	Young adults	Old adults
Age (years)	23.2 ± 2.7	65.8 ± 4.5
Sex (male; female)	10; 10	9; 10
Height (m)	1.79 ± 0.10	1.72 ± 0.10
BMI (kg/m^2^)	22.1 ± 2.5	26.0 ± 3.1
SPPB score	12.0 ± 0.0	11.8 ± 0.5
MMSE score	29.9 ± 0.5	28.8 ± 1.9
SQUASH		
Total score	11,343 ± 5156	10,019 ± 3900
Light (min/w)	1823 ± 899	1337 ± 1052
Moderate (min/w)	526 ± 373	503 ± 378
Heavy (min/w)	349 ± 312	564 ± 443

Total score is minutes per week × intensity of the activity. The amount of light, moderate, and heavy exercises is expressed in minutes per week

*BMI* body mass index, *SPBB* short physical performance battery (max. score of 12), *MMSE* mini mental state examination (max. score of 30), *SQUASH* short questionnaire to assess health-enhancing physical activity

### Experimental procedures

Subjects were instructed to stand upright on two force plates (Bertec 4060-08, Columbus, OH, USA), wearing comfortable shoes without high heels. With the arms placed parallel to the body, subjects looked at a “+” sign displayed on a projection screen. Although there were no specific instructions on initial foot placement, markings on the force platform around the shoes ensured consistent foot positioning throughout the experiments (intermalleolar distance, young: 17 ± 0.9 cm, old: 14 ± 0.9 cm). The CoP signal was sampled at 100 Hz and filtered using the fourth-order low-pass Butterworth filter with a cut-off frequency of 10 Hz.

**Fig. 1:**
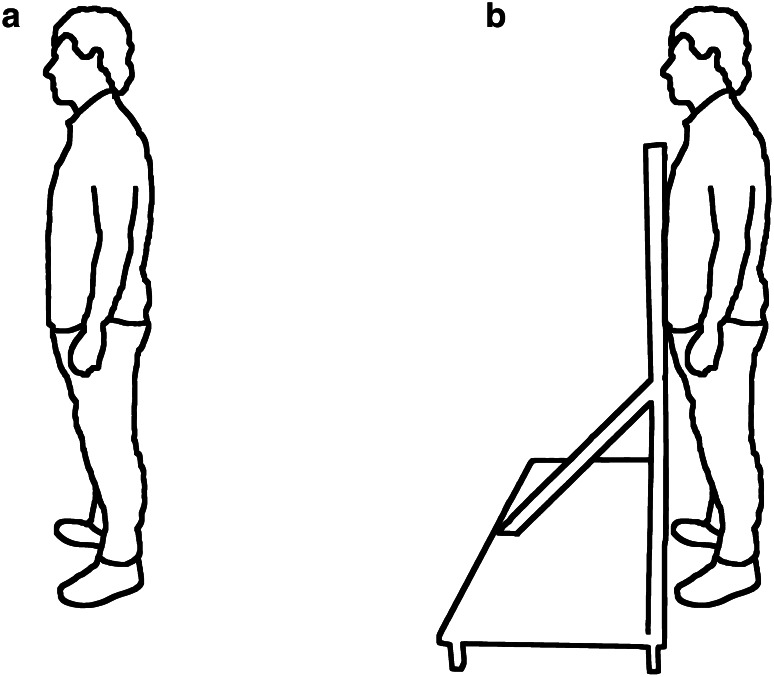
Illustration of **a** unsupported and **b** supported standing conditions

TMS and PNS were applied separately during two standing conditions: unsupported and supported standing (Fig. 1). The order of condition and stimulation type was randomized between subjects. During unsupported standing, participants stood naturally upright. During supported standing, participants stood upright and were asked to remain lightly in contact with a wooden board at the chest without leaning against it. The position of the board was adjusted for each participant, so that their CoP position was similar between conditions.

### EMG

Because of a technical malfunction, surface electromyography (EMG) of the right soleus muscle and tibialis anterior was recorded using two different systems from the same company (young adults: model Bagnoli-8, old adults: Trigno™ Wireless System, Delsys, Natick, MA, USA). Active electrodes (young adults: DE-2.1, old adults: Trigno wireless EMG sensor) were placed over the muscle belly, and in young adults, a reference electrode was placed on the medial aspect of the tibia. The EMG signal was amplified 1000×, sampled at 5 kHz (young adults) or 4 kHz (old adults), and bandpass filtered with the second-order Butterworth filter (10–1000 Hz) using data acquisition interface and software (Power 1401 and Signal 5, Cambridge Electronics Design, Cambridge, UK). To get a measure of the maximum EMG value, subjects performed a maximum voluntary contraction (MVC) of the soleus and tibialis anterior for 5 s. For the soleus, subjects were standing on the toes with resistance from a strap attached to a harness worn by the subjects. For the tibialis anterior, manual resistance was given by the experimenter, while subjects were seated in a chair with the knee in 45° flexion and the ankle in neutral position. The MVC was quantified as the maximum root mean square value (50-ms window). The background EMG in a 50-ms window before every TMS pulse was rectified, averaged, and expressed as a percentage of MVC.

### Behavioral data acquisition and analysis

A 2-s window before every TMS pulse was used for CoP data analysis. This window was chosen, so that the CoP measurement was close to the TMS measurement, but not influenced by the postural perturbation caused by the previous TMS pulse. CoP position and velocity in the anteroposterior direction were calculated for each of these time periods and, then, averaged across time periods. CoP velocity is a reliable (Demura et al. 2008) and discriminative index of body sway (Raymakers et al. 2005).

### PNS data acquisition and analysis

The tibial nerve of the right leg was stimulated in the popliteal fossa using a constant-current stimulator (Digitimer DS 7, Hertfordshire, UK). The stimulating electrode (bipolar, cathode proximal) was fixed tightly with a Velcro strap around the leg. A recruitment curve was assembled during supported standing to determine the stimulation intensity required for the experimental trials. We increased stimulation intensity in steps of 0.5 mA until the M-wave amplitude in the soleus no longer increased. When the M-wave ceased to increase and plateaued, stimulation intensity was further increased with 20 % to ensure that the maximal M-wave was obtained. Stimulation intensity during the experiment was set at the intensity that evoked a response of 50 % of the maximal H-reflex amplitude on the ascending part of the recruitment curve. Using this stimulation intensity, 20 H-reflexes were evoked during supported and unsupported standing. To reduce variability in the H-reflex (Tokuno et al. 2009), PNS was triggered only when CoP moved forward, as assessed online using CoP velocity, and with a minimal interval of 5 s between trials. H-reflex and M-wave amplitude were expressed as a percentage of the M-max.

### TMS data acquisition and analysis

Transcranial magnetic stimuli were delivered to the cranium over the left M1 with a double cone coil (inner loop diameter 110 mm) connected to a Magstim 2002 and Bistim2 (Magstim, Whitland, UK). The current flowed from the anterior to posterior direction in the coil. The optimal location for eliciting motor evoked potentials (MEPs) with the largest amplitude at a given intensity in the soleus of the right leg was determined by moving the coil systematically in steps of 0.5 cm over M1 area starting at the vertex. The final location was marked on the scalp with a permanent marker to enable the experimenter to hold the coil at a consistent stimulation location throughout the experiment. We determined the motor threshold (MT) in the supported standing condition. MT was the lowest intensity at which the MEPs in the soleus were larger than 100 μV in at least three out of five consecutive trials (Beck et al. 2007; Taube et al. 2011; Tokuno et al. 2009). Stimulation intensity of the conditioning and test pulse was set at 0.8 and 1.2 MT, respectively. As recommended by Garry and Thomson (2009), we did not adjust the stimulation intensity between conditions, as SICI is influenced by stimulation intensity rather than test MEP size (Garry and Thomson 2009; Zoghi and Nordstrom 2007). Paired-pulse TMS with an interstimulus interval of 2.5 ms was used to assess SICI, while an interstimulus interval of 13 ms was used to assess ICF. In preliminary studies, we found these intervals to produce the largest SICI and ICF, respectively, consistent with the literature (Soto et al. 2006). In both conditions, there were ten test MEP, ten SICI, and ten ICF trials, presented in a randomized order. Stimuli were only given in the forward phase of sway and with a minimal interval of 5 s between trials.

MEP size was quantified by calculating the peak-to-peak amplitude. SICI and ICF were expressed as percentage inhibition and facilitation, by using the following formula for SICI: 100 − (conditioned MEP/test MEP × 100) and the following formula for ICF: (conditioned MEP/test MEP × 100) − 100. Although stimulation location and intensity were set for the soleus, in 15 young and 11 old subjects, we managed to concurrently record consistent MEPs in the tibialis anterior. Therefore, data from the tibialis anterior are also presented.

### Statistical analysis

All variables were checked for Gaussian distribution prior to analysis. An independent sample *t* test was used to check differences in MT between young and old adults. CoP position, CoP velocity, background EMG, H-reflex amplitude, M-wave amplitude, test MEP amplitude, SICI, and ICF were analyzed using an age (young and old) by condition (supported standing and unsupported standing) ANOVA with repeated measure on condition. A series of covariance analysis were conducted to test whether significant condition effects were confounded by differences between conditions in background EMG, test MEP amplitude, and CoP position. Significant age effects were tested for possible confounders using Pearson correlation. All statistical analyses were conducted using SPSS 17.0 (Chicago, IL, USA). The alpha level was set at 0.05. Data are presented as mean ± SD.

## Results

### Center of pressure

**Fig. 2:**
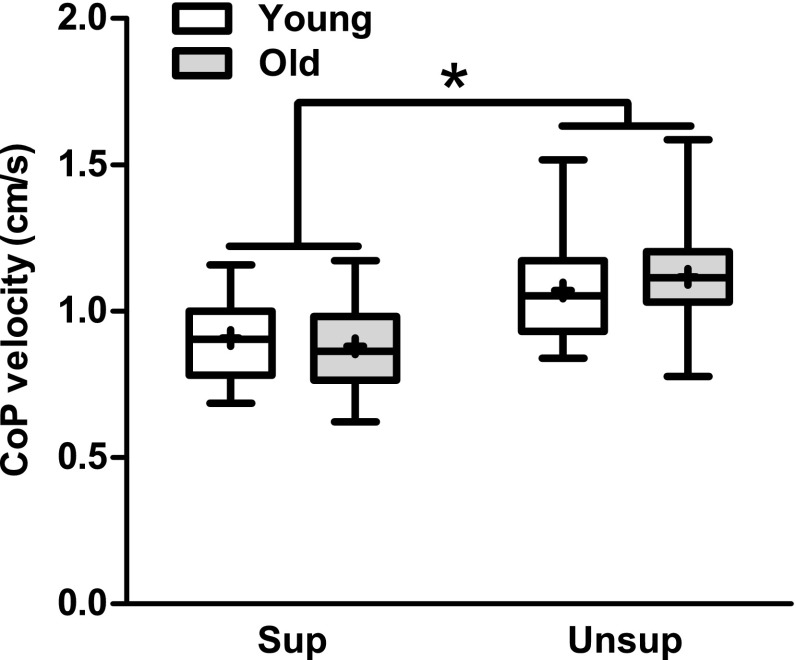
Group data for young and old adults’ center of pressure velocity in the anteroposterior direction when standing supported (sup) and unsupported (unsup), showing a significant condition effect (*p* < 0.001). The *horizontal line* within the *box* indicates the median value, the *box* covers the 25th–75th percentiles, and the *whiskers* represent the range

Mean CoP position was 1.5 ± 0.3 cm more forward (*F*_1,25_ = 29.2, *p* < 0.001), and CoP velocity was 23 ± 12 % greater (*F*_1,25_ = 21.1, *p* < 0.001) during unsupported compared with supported standing (Fig. 2). There were no age (position: *F*_1,25_ = 1.5, *p* = 0.229; velocity: *F*_1,25_ = 0.4, *p* = 0.397) or age by condition interaction effects (position: *F*_1,25_ = 0.1, *p* = 0.727; velocity: *F*_1,25_ = 0.5, *p* = 0.492).

### Background EMG

**Fig. 3:**
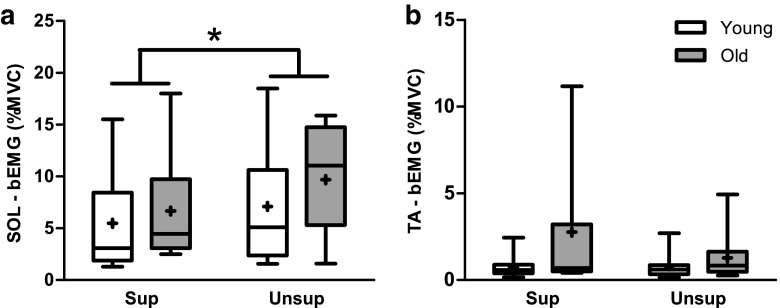
Group data for young and old adults of background EMG in **a** the soleus muscle (condition effect, *p* < 0.001) and **b** the tibialis anterior muscle (interaction effect, *p* = 0.035). *MVC* maximum voluntary contraction. The *horizontal line* within the *box* indicates the median value, the *box* covers the 25th–75th percentiles, and the *whiskers* represent the range

There was a significant condition effect (*F*_1,26_ = 31.6, *p* < 0.001) in background EMG in the soleus muscle (Fig. 3a), with ~40 % higher muscle activity in unsupported vs. supported standing. There was, however, no age effect (*F*_1,26_ = 0.9, *p* = 0.351) or age by condition interaction (*F*_1,26_ = 2.3, *p* = 0.145). For the tibialis anterior background EMG, there were no age and condition effects, although there was a trend for greater background EMG in old vs. young adults (*F*_1,24_ = 4.0, *p* = 0.057) and during supported vs. unsupported standing (*F*_1,24_ = 4.2, *p* = 0.052) (Fig. 3b). There was an age by condition interaction (*F*_1,24_ = 5.0, *p* = 0.035), with the background EMG being almost twice as high in supported vs. unsupported standing in old but not in young adults. Note that the overall background EMG level in the tibialis anterior was only 0.8 and 2.0 % of MVC in young and old adults, respectively, suggesting that it had minimal, if any, effects on SICI and ICF.

### PNS measures

There was no significant difference in the M-max amplitude between young and old adults (young: 3.7 ± 1.3 mV; old: 3.0 ± 1.6 mV; *t*_31_ = 0.171). There were also no significant differences in the H-reflex amplitude between age groups (young: 17 ± 1 % of M-max; old: 16 ± 2 % of M-max) or support conditions (supported: 16 ± 1 % of M-max; unsupported: 17 ± 2 % of M-max) (*F*_1,31_ < 0.1, *p* = 0.807; *F*_1,31_ = 2.8, *p* = 0.105). In 10 young and 13 old subjects, the H-reflex was accompanied by an M-wave. M-wave amplitudes were similar in the two age groups (young: 8 ± 2 % of M-max; old: 15 ± 3 % of M-max; *F*_1,21_ = 1.7, *p* = 0.212) and two standing conditions (supported: 13 ± 3 % of M-max; unsupported 11 ± 3 % of M-max; *F*_1,21_ = 0.6, *p* = 0.465).

### TMS measures in the soleus

MT was similar in young (48 ± 5 % of maximal stimulator output, range 40–55 %) and old (51 ± 6 %, range 38–60 %) adults (*t*_26_ = −1.7, *p* = 0.101), resulting in similar stimulation intensities (1.2*MT). Old vs. young adults tended to have greater test MEP amplitude recorded in the soleus (old: 0.34 ± 0.19 mV; young: 0.23 ± 0.12 mV; *F*_1,26_ = 3.8, *p* = 0.063). The test MEP increased from supported (0.27 ± 0.17 mV) to unsupported (0.31 ± 0.16 mV) standing (*F*_1,26_ = 5.9, *p* = 0.022).

**Fig. 4:**
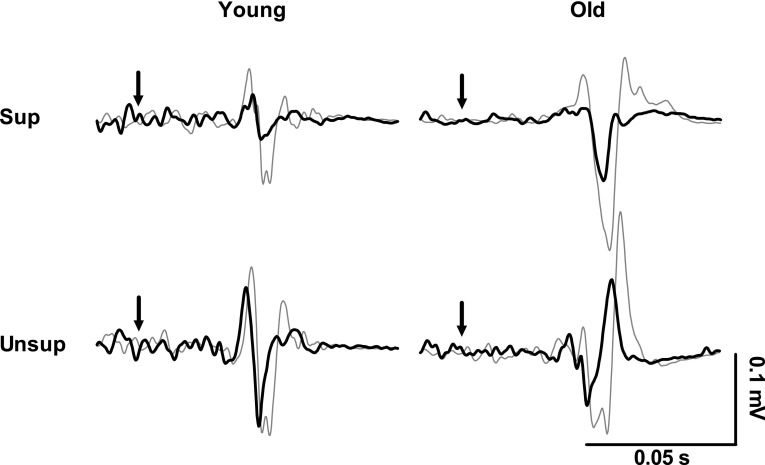
Representative responses to transcranial magnetic brain stimulation in the soleus muscle of one 23-year-old male and one 62-year-old male subject while standing supported (sup) and unsupported (unsup). Waveforms represent the average of 10 motor evoked potentials in response to an unconditioned test pulse (*thin gray line*) and to a conditioned test pulse (*thick black line*) given at an interpulse interval of 2.5 ms. *Black arrows* indicate the time when the test pulse is given. Note the down-modulation of short interval intracortical inhibition (SICI) when standing unsupported vs. supported in both young and old subjects

**Fig. 5:**
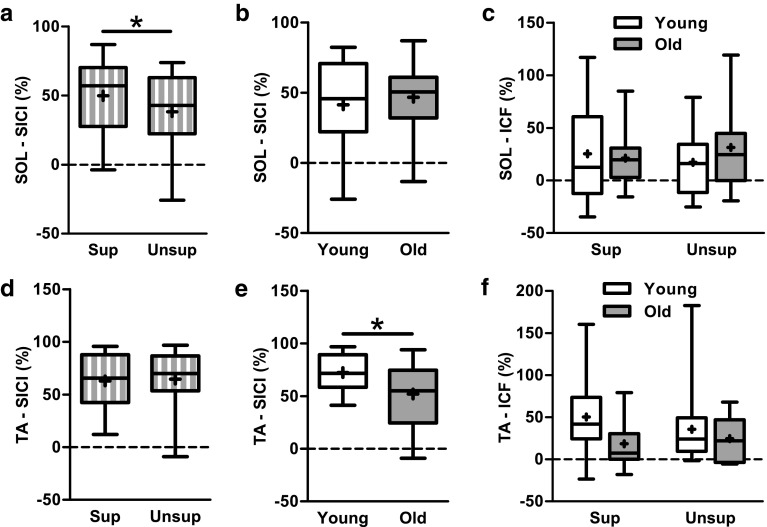
Group data for **a** short interval intracortical inhibition (SICI) in the soleus during supported and unsupported standing (condition effect, *p* = 0.007), **b** SICI in the soleus in young and old adults (no age effect), **c** intracortical facilitation (ICF) in the soleus, **d** SICI in the tibialis anterior during supported and unsupported standing (no condition effect), **e** SICI in the tibialis anterior in young and old adults (age effect, *p* = 0.030), and **f** ICF in the tibialis anterior muscle. Greater values for SICI and ICF represent more inhibition and facilitation, respectively. *Sup* supported and *unsup* unsupported. The *horizontal line* within the *box* indicates the median value, the *box* covers the 25th–75th percentiles, and the *whiskers* represent the range

Figure 4 shows the effect of standing support on TMS responses in the soleus muscle of a representative young and old subject. Across all subjects, age did not affect SICI (*F*_1,26_ = 0.3, *p* = 0.591), but SICI was 30 % lower in unsupported compared with supported standing (*F*_1.26_ = 8.6, *p* = 0.007) (Fig. 5a, b). Age and condition had no effect on ICF (*F*_1,25_ = 0.2, *p* = 0.679; *F*_1,25_ = 0.02, *p* = 0.885) (Fig. 5c). There were no age by condition interaction in any of the TMS measures (test MEP: *F*_1,26_ = 0.4, *p* = 0.532; SICI: *F*_1,26_ = 0.3, *p* = 0.574; ICF: *F*_1,25_ = 1.3, *p* = 0.258).

To determine if potential confounders affected our main results, we conducted a series of covariance analyses. When the difference in background EMG between the two standing conditions was added as a covariate in the analysis, the condition effect on test MEP amplitude was no longer significant (*F*_1,25_ < 0.1, *p* = 0.879). However, the condition effect on SICI remained significant when adding differences between conditions in background EMG (*F*_1,25_ = 5.1, *p* = 0.033), test MEP size (*F*_1,25_ = 8.6, *p* = 0.007), and CoP position (*F*_1,24_ = 4.5, *p* = 0.045), respectively.

### TMS measures in the tibialis anterior

In the tibialis anterior, test MEP size was greater in old (0.72 ± 0.33 mV) than in young (0.36 ± 0.29 mV) adults (*F*_1,24_ = 9.7, *p* = 0.005) but was similar in supported (0.52 ± 0.38 mV) and unsupported (0.50 ± 0.34 mV) standing (*F*_1,24_ = 0.6, *p* = 0.441). In contrast to the soleus, there was ~30 % less SICI in old vs. young adults (*F*_1,24_ = 5.3, *p* = 0.030) (Fig. 5d, e), but SICI was similar between standing conditions (*F*_1,24_ = 0.3, *p* = 0.597). Age and condition did not affect ICF in the tibialis anterior (age: *F*_1,23_ = 2.2, *p* = 0.155; condition: *F*_1,23_ = 0.5, *p* = 0.510) (Fig. 5f). There were no age by condition interaction in any of the TMS measures (test MEP: *F*_1,24_ = 1.9, *p* = 0.181; SICI: *F*_1,24_ = 0.1, *p* = 0.822; ICF: *F*_1,23_ = 3.0, *p* = 0.099). There was no correlation between test MEP size and SICI (young: *r* = 0.07, *p* = 0.701; old: *r* = −0.18, *p* = 0.418), showing that the age effect in SICI was not due to the age effect in test MEP size.

## Discussion

Recently, we have shown an age-related reduction in SICI with an increase in balance task demand (Papegaaij et al. 2014). This study extends these previous findings by determining whether the age-related difference in modulation of intracortical circuits is also present when switching from a non-balance task (i.e., supported standing) to a relatively simple balance task, i.e., normal standing on rigid surface. The present results revealed a task-related modulation of SICI independent of age, highlighting the importance of task difficulty on age-related differences in SICI. An additional new finding is that the age effects on SICI may be muscle-specific, as age affected SICI in the tibialis anterior but not in the soleus muscle. Neither age nor standing condition affected the H-reflex amplitude in the soleus.

### Posture-related modulation of SICI in young and old adults

SICI in the soleus was lower in both age groups in the unsupported compared with supported condition. Reductions in SICI in relation to motor tasks have been reported previously, for example, during movement preparation (Heise et al. 2013) and during the activation phase compared with the deactivation phase in cycling (Sidhu et al. 2013). In a previous study, we found a decrease in SICI in old but not in young adults when standing on foam vs. standing on a rigid surface (Papegaaij et al. 2014). We argued that the change from rigid to foam surface challenged old compared with young subjects more, reflected by the 47 and 20 % increase in sway velocity in old and young subjects, respectively (Papegaaij et al. 2014). In this study, changes in sway velocity were comparable between young and old participants when switching from supported to unsupported standing. Thus, it seems that elderly subjects have similar strategies to adapt SICI when considering simple, non-challenging balance tasks. In contrast, when postural demands are high, the threshold for disinhibition seems to be lower (Papegaaij et al. 2014). Possibly, a decrease in SICI heightens the state of readiness in M1 and prepares it to become more easily activated or to activate other neural structures on demand. This interpretation fits well with the current data concerning a switch from a non-balance to a simple balance task: a reduction in SICI would set M1 and perhaps related motor structures in a state that would allow more effective responses to potential balance threats. An alternative interpretation is that SICI is reduced to activate the muscles around the ankle and ensure ankle stiffness. However, this seems unlikely, as the modulation of SICI was not related to changes in background EMG, a finding discussed below.

We considered the impact of potential confounders on our main finding, i.e., postural modulation of SICI. Covariance analyses showed that test MEP size, CoP position, and background EMG did not affect the SICI modulation between standing conditions. The condition effect on test MEP size, however, was mediated by the increased level of background EMG. These results are supported by a subanalysis of the subjects who had only minor changes in background EMG between conditions (0.3 ± 1.4 % of MVC increase from supported to unsupported standing). In this subgroup of eight subjects (five young, three old), half increased and half decreased their test MEP amplitude from supported to unsupported standing. On the contrary, seven out of the eight subjects decreased their SICI. In addition, the average decrease in SICI is similar in this subgroup (29 %) compared with the rest of the subjects (30 %). Our protocol of not adjusting test MEP size between conditions is strongly supported by previous data, showing that SICI should be examined using constant test TMS intensity regardless of changes in test MEP size (Garry and Thomson 2009). Therefore, the modulation in SICI was related to the switch from a non-balance task to a balance task and not to differences in background EMG or MEP size.

Interestingly, the SICI modulation was muscle-specific, as young and old adults did not show any modulation in SICI when measured in the tibialis anterior but did show modulation with standing conditions when measured in the soleus muscle. This observation is consistent with data from Soto et al. (2006), showing a reduction in SICI in the soleus but not in the tibialis anterior when standing compared with rest, and agrees with the role of the plantar flexors as being the agonistic muscles for standing (Di Giulio et al. 2009).

### Do age-related changes in SICI depend on the muscle?

In this study, there were no age-related changes in SICI in the soleus muscle. In our previous study, we found lower SICI in old compared with young adults in the tibialis anterior (Papegaaij et al. 2014). These results might signify that age-related changes are muscle-specific. Indeed, the data that we obtained from the tibialis anterior in this study were consistent with the data from our previous study, showing an age-related reduction in SICI. This muscle-specificity may be related to different neural circuits controlling the soleus and tibialis anterior muscles. A functional magnetic resonance imaging study in healthy young adults reported that dorsal flexion evoked extensive brain activation in motor cortical areas, whereas plantar flexion mainly activated subcortical structures (Trinastic et al. 2010). Moreover, motor unit recordings after single TMS pulses showed that corticospinal projections to the soleus muscle were weaker than those to the tibialis anterior (Brouwer and Ashby 1992). Additional evidence supporting the hypothesis of a specific age-related reduction in SICI for muscles with strong corticospinal projections comes from muscles of the upper extremity. When examined in hand muscles, reduced (Heise et al. 2013; Marneweck et al. 2011) or similar (Oliviero et al. 2006; Smith et al. 2011) SICI has been reported in old compared with young adults. However, when examined in wrist flexors and extensors, which have weaker monosynaptic projections, SICI was greater in middle-aged and old adults (Kossev et al. 2002; McGinley et al. 2010). This study is the first to demonstrate this age by muscle interaction within a study and in relation to upright standing.

### No age- or posture-related changes in ICF

Consistent with our previous study (Papegaaij et al. 2014), we found no age- or posture-related changes in ICF. This may indicate that modulating the descending drive to lower limb muscles during upright standing depends on up- or down-regulation of intracortical inhibition (disinhibition) and not facilitation. We speculate that reducing inhibition may be preferred over increasing facilitation to limit induced noise. However, we cannot exclude that other facilitatory neural circuits that we did not measure were modulated between postures.

### No age- or posture- related changes in H-reflex

In this study, there were no age- or posture- related changes in H-reflex amplitude. This is inconsistent with Tokuno et al. (2009) and Baudry et al. (2014b), who reported the reduced H-reflex amplitude during unsupported compared with supported standing. Furthermore, most previous studies reported reduced H-reflex with increased age and balance task difficulty (Angulo-Kinzler et al. 1998; Koceja et al. 1995). As the methodology used in previous studies is comparable with ours, it is unclear what caused this inconsistency. One possible explanation is that we had a greater increase in background EMG between standing support conditions (40 %) compared with the other studies (Tokuno et al.: 9 %, Baudry et al.: 5 %). This increase in background EMG may have facilitated the H-reflex size and, therefore, counteracted the potential decrease due to balance task difficulty. A decreased H-reflex combined with an increased corticospinal or intracortical excitability would suggest an increased descending drive to control leg muscles during upright stance (Baudry et al. 2014b). However, the current results can neither confirm nor disprove this hypothesis.

### Clinical implication of the results

The current and previous data are compatible with the concept of prescribing exercise training that includes balance tasks with increasing difficulty for old adults. Task difficulty, perhaps a proxy for ‘intensity’ of balance training, may be an important element of these exercise programs and should possibly be relatively high and incremental to cause favorable neural adaptations (Sherrington et al. 2008). Future studies will verify this suggestion, because, currently, there are no studies that report changes in M1 intracortical circuits after balance training in old adults.

### Limitations of the study

We recorded responses in one muscle (tibialis anterior) to TMS while actually targeting another muscle (soleus), a method used previously (Soto et al. 2006; Yamaguchi et al. 2012). Although this method has its limitations, generally, the threshold of the tibialis anterior is lower compared with the soleus, so that many subjects produce reliable responses in the tibialis anterior to suprathreshold TMS targeting the soleus, and these responses are not the result of cross talk but instead are muscle-specific responses to the stimulation (Geertsen et al. 2010; Hansen et al. 2005). Moreover, the degree of inhibition and the age-related differences in this study (young: 73 ± 5 %, old: 53 ± 9 %) were comparable with our previous study (young: 67 ± 8 %, old: 44 ± 5 %) where we did target the tibialis anterior (Papegaaij et al. 2014), suggesting that the tibialis anterior data in this study are in all likelihood reliable. Another limitation is that most participants were relatively fit and physically active, shown by the high SPPB and SQUASH scores. Therefore, the results are relevant to a fit segment of seniors and may not be generalizable to less active individuals, in whom an age-effect could be more prominent.

### Conclusions

This study demonstrates that intracortical inhibition in the soleus muscle decreased when subjects changed from a non-balance to a balance task in which the nervous system needs to control body sway. In contrast to the results of our previous study that involved much more challenging balance tasks and, therefore, highlighting the importance of task difficulty, the modulation of SICI was independent of age. Moreover, the present results support the hypothesis of a specific age-related reduction in SICI preferentially for muscles with strong corticospinal projections. Overall, a combination of past and current data suggests that the M1 is involved in standing control and that its role becomes more prominent with an increase in task difficulty and age.

## Abbreviations

CoP: Center of pressure
EMG: Electromyography
H-reflex: Hoffmann reflex
ICF: Intracortical facilitation
M1: Primary motor cortex
MEP: Motor-evoked potential
MMSE: Mini mental state examination
MT: Motor threshold
MVC: Maximum voluntary contraction
PNS: Peripheral nerve stimulation
SICI: Short-interval intracortical inhibition
SPPB: Short physical performance battery
SQUASH: Short questionnaire to assess health-enhancing physical activity
TMS: Transcranial magnetic stimulation
